# Computed tomography angiography-guided conservative management of a penetrating ballistic neck injury involving the carotid sheath: A case report

**DOI:** 10.1016/j.radcr.2025.10.008

**Published:** 2025-11-11

**Authors:** Imane Boulahroud, Dounia Haida, Imane Halaouate, Safae Elyaalaoui, Mehdi Lekehal, Asma Jdar, Ayoub Bounssir, Tarik Bakkali, Brahim Lekehal

**Affiliations:** a Department of Vascular Surgery, CHU Ibn Sina, Rabat, Morocco; b Department of Vascular Surgery, Mohammed V Military Instruction Hospital, Rabat, Morocco

**Keywords:** Penetrating neck injury, Common carotid artery, Computed tomography angiography, Conservative management, Ballistic injury, Case report

## Abstract

Penetrating ballistic neck injury carries a high risk of vascular injury, particularly to the common carotid artery (CCA), due to its superficial location and critical role in cerebral perfusion. While surgical intervention is indicated in the presence of “hard signs” of vascular injury, the role of conservative management in asymptomatic patients remains debated. We report the case of a 45-year-old male who sustained a penetrating ballistic injury while seated and cleaning his 5-shot hunting rifle (shotgun). The accidental discharge occurred at less than 1 meter, producing a short-range, low-velocity dispersion of 42 metallic pellets across the craniofacial, cervical, and thoracoabdominal regions. The trajectory was oblique, consistent with the patient’s seated position. Computed tomography angiography (CTA) revealed multiple pellets adjacent to the carotid sheath without evidence of vascular disruption, active bleeding, or neurological deficit. The patient was managed conservatively with close clinical and imaging surveillance. This case highlights the feasibility and safety of nonoperative management in carefully selected, hemodynamically stable patients with penetrating ballistic cervical injury, even when projectiles are located near major vascular structures. CTA was pivotal for initial assessment, surgical decision‑making, and follow‑up planning. A structured surveillance protocol incorporating serial neurological examinations and repeat imaging is proposed to detect delayed complications such as pseudoaneurysm, thrombosis, or dissection. In the absence of clinical or radiological signs of vascular injury, CTA-guided conservative management can be a safe alternative to surgery, provided rigorous monitoring is ensured. This approach may reduce unnecessary surgical morbidity while maintaining patient safety.

## Introduction

Penetrating ballistic neck injuries pose a significant diagnostic and therapeutic challenge due to complex projectile trajectories and the potential for simultaneous damage to multiple anatomical regions [1]. The cervical region is particularly vulnerable, with the common carotid artery (CCA) at high risk because of its superficial location and critical role in cerebral perfusion.

Cervical vascular injuries may result in immediate complications such as massive hemorrhage, cerebral ischemia, and airway compromise, as well as delayed sequelae including pseudoaneurysm, thrombosis, and arterial dissection. These risks demand meticulous and timely evaluation [1].

Computed tomography angiography (CTA) has become the cornerstone of initial assessment in penetrating neck trauma, allowing precise localization of projectiles and detailed evaluation of vascular integrity, thereby guiding early management decisions.

We report an exceptional case of a hunting‑related shotgun injury with multiple pellets lodged in proximity to the carotid sheath, managed entirely conservatively. This case demonstrates that, in carefully selected asymptomatic patients, non‑operative management can be both feasible and safe. We also propose a structured surveillance protocol aligned with current evidence‑based recommendations.

## Case presentation

A 45-year-old male farmer, with no significant past medical or surgical history apart from active smoking, presented to the emergency department 2 hours after an accidental discharge of a 5-shot hunting rifle (shotgun) while he was seated and cleaning the weapon. The shot occurred at less than 1 meter, producing a short-range, low-velocity dispersion pattern. The general trajectory was oblique, consistent with the patient’s seated position at the time of the accident. He reported odynophagia and right eyelid hematoma without visual loss.

**Fig. 1:**
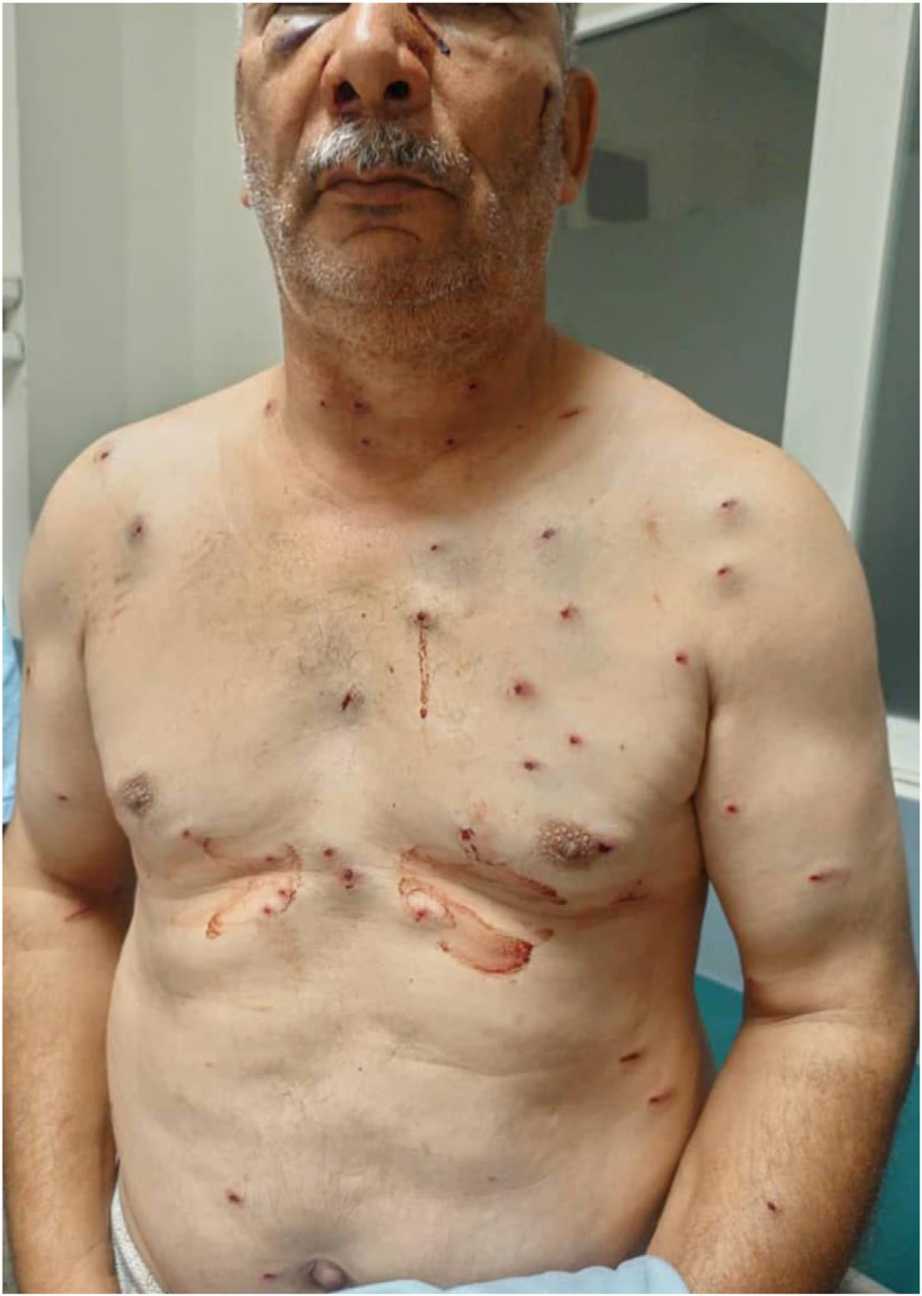
Clinical photograph showing the distribution of 42 metallic pellets from a hunting‑related shotgun injury, involving the neck, thorax, abdomen, and upper limbs.

On examination, the patient was hemodynamically, respiratory and neurologically stable. Multiple punctate entry wounds (42 pellets) were noted over the neck, thorax, abdomen and upper limbs (Fig. 1). Ocular examination revealed conjunctival hyphemia with eyelid edema. There was no expanding hematoma, active bleeding, stridor, facial asymmetry, or neurological deficit.

Given the patient’s stable condition, whole‑body multidetector computed tomography angiography (CTA) was performed, revealing 42 metallic pellets distributed across multiple anatomical regions:

### Craniofacial and intracranial findings

• Metallic projectile adjacent to the right maxilla.
• Ipsilateral eyelid hematoma.
• Simple fracture of the inferior wall of the right orbit.
• Left lateral peri‑ocular projectile in contact with the medial rectus muscle, without intraocular injury.
• Brain: no abnormal parenchymal density, no peri-cerebral collection, preserved midline structures, and normal cisterno‑ventricular system.

### Cervical findings

**Fig. 2:**
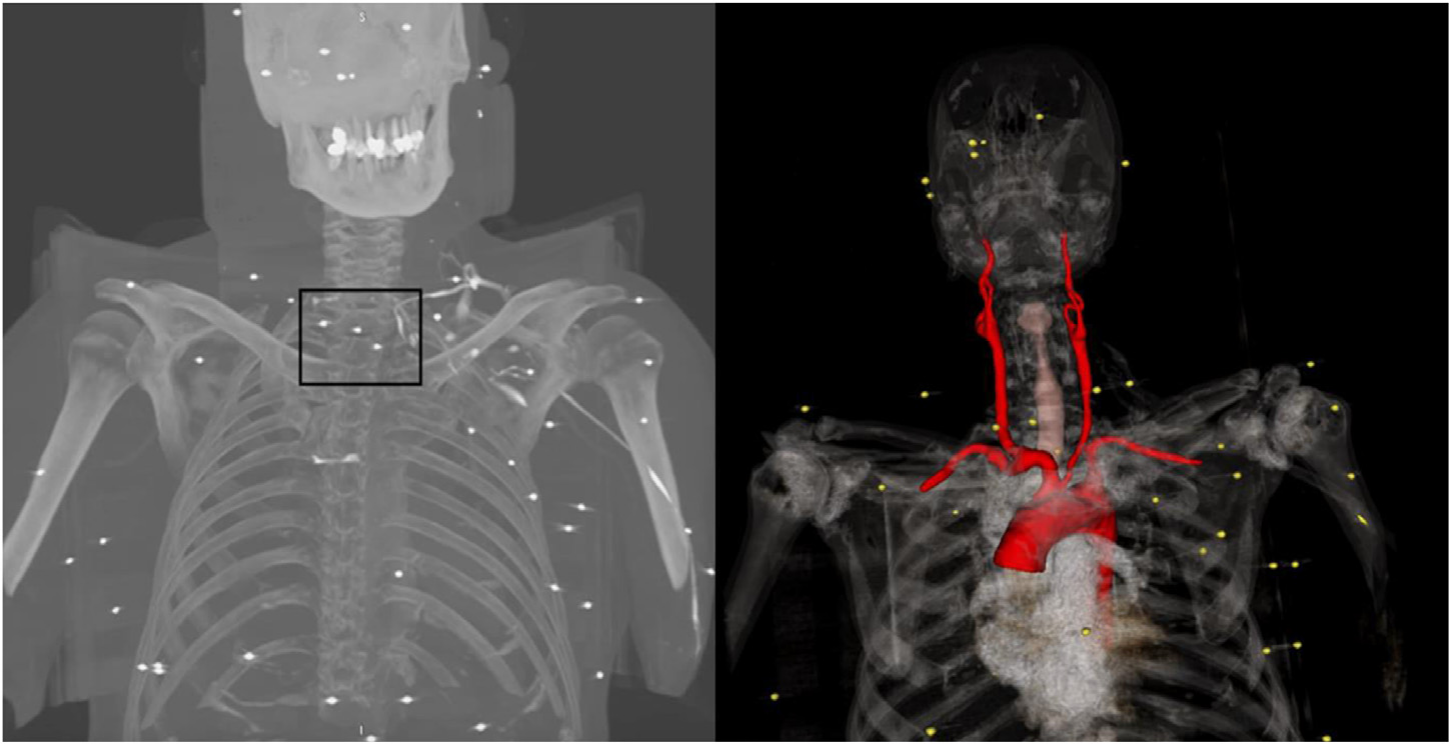
Computed tomography angiography (CTA) image demonstrating multiple metallic pellets distributed across the neck, thorax, and upper limbs.

**Fig. 3:**
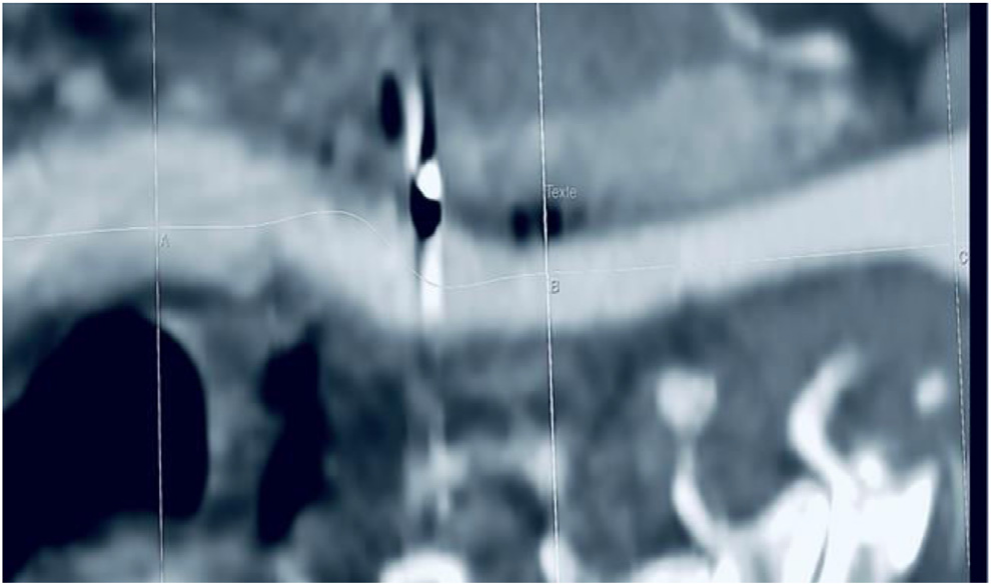
CTA centerline reconstruction of the right common carotid artery demonstrating a metallic pellet trajectory in close contact with the vessel wall, without evidence of luminal narrowing or contrast extravasation.

**Fig. 4:**
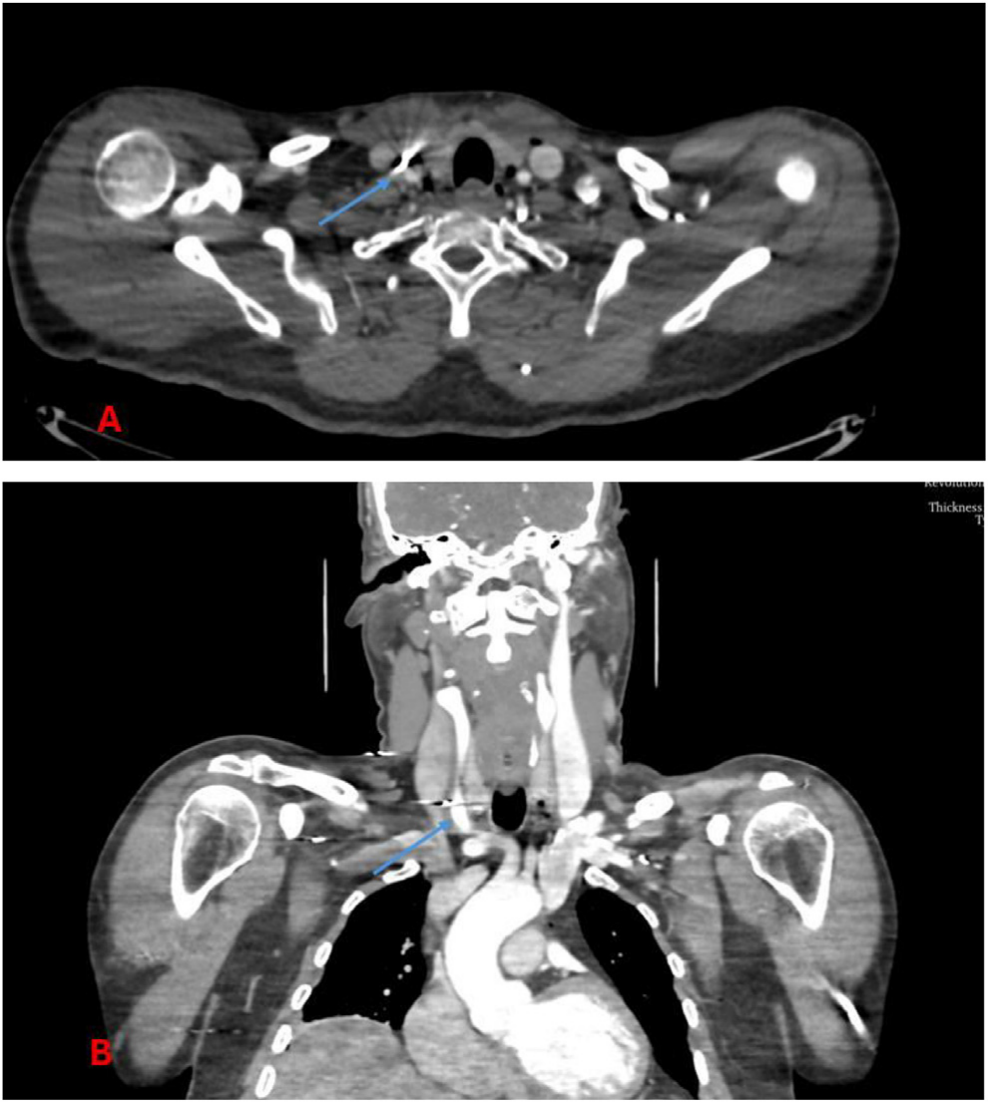
CTA image of the neck demonstrating a metallic pellet within the right common carotid sheath (arrow), without evidence of luminal narrowing or contrast extravasation. (A) axial view; (B) coronal view.
• Pretracheal, in contact with the right thyroid lobe, without visible tracheal breach (Fig. 5).

Four projectiles:

• Situated anterolaterally to the right carotid sheath, in direct contact with the common carotid artery, without evidence of perivascular hematoma (Fig. 2, Fig. 3, Fig. 4).

**Fig. 5:**
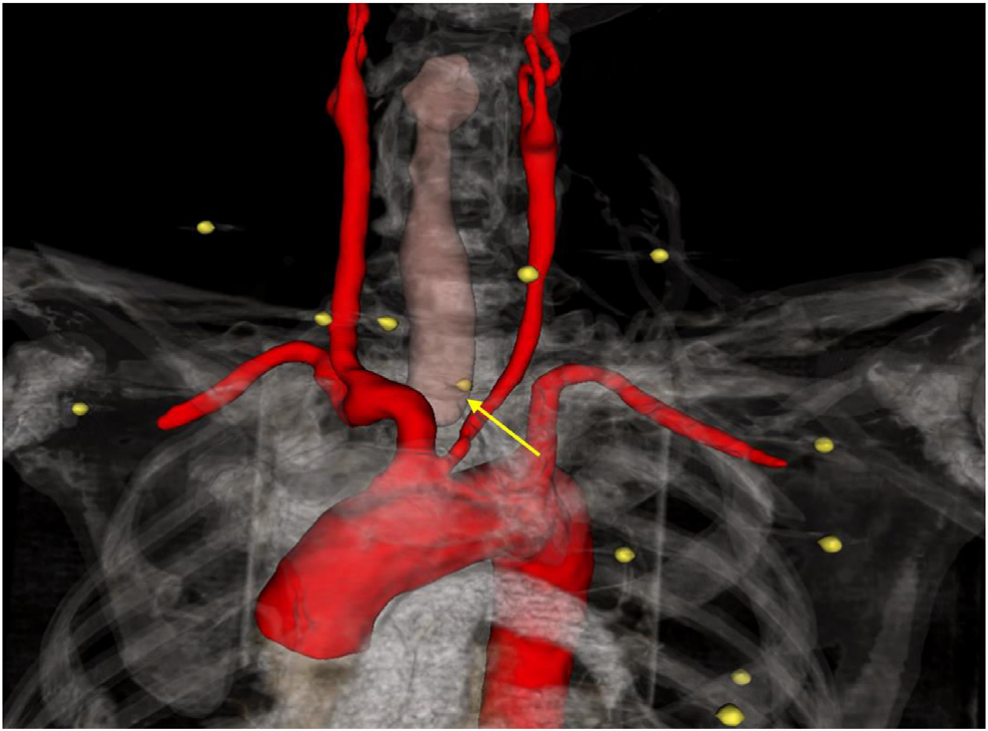
CTA image of the neck demonstrating a metallic pellet adjacent to the trachea (yellow arrow).
• Post‑tracheal, anterior to the esophagus, without direct perforation.
• Associated finding: superior pneumomediastinum, suggesting a possible high aerodigestive tract breach.

### Thoracic findings

• Anterior intrathoracic projectile adjacent to the left upper lobe, with focal pulmonary contusion.
• Intramuscular projectile in the right first intercostal space.
• Intermuscular projectile between the left pectoral muscles.
• Minimal right pneumothorax without mediastinal shift.
• Abdominopelvic findings.
• Intramuscular projectile in the right rectus abdominis muscle.
• Subcutaneous projectiles, including 1 in the peri‑umbilical region.
• Normal liver and no visceral injury.

### Management

Based on multidisciplinary agreement involving vascular and thoracic surgeons, a conservative management strategy was implemented. The patient was admitted to the intensive care unit for continuous monitoring, received analgesia and antibiotic prophylaxis, and underwent ophthalmologic assessment as part of comprehensive care.

**Fig. 6:**
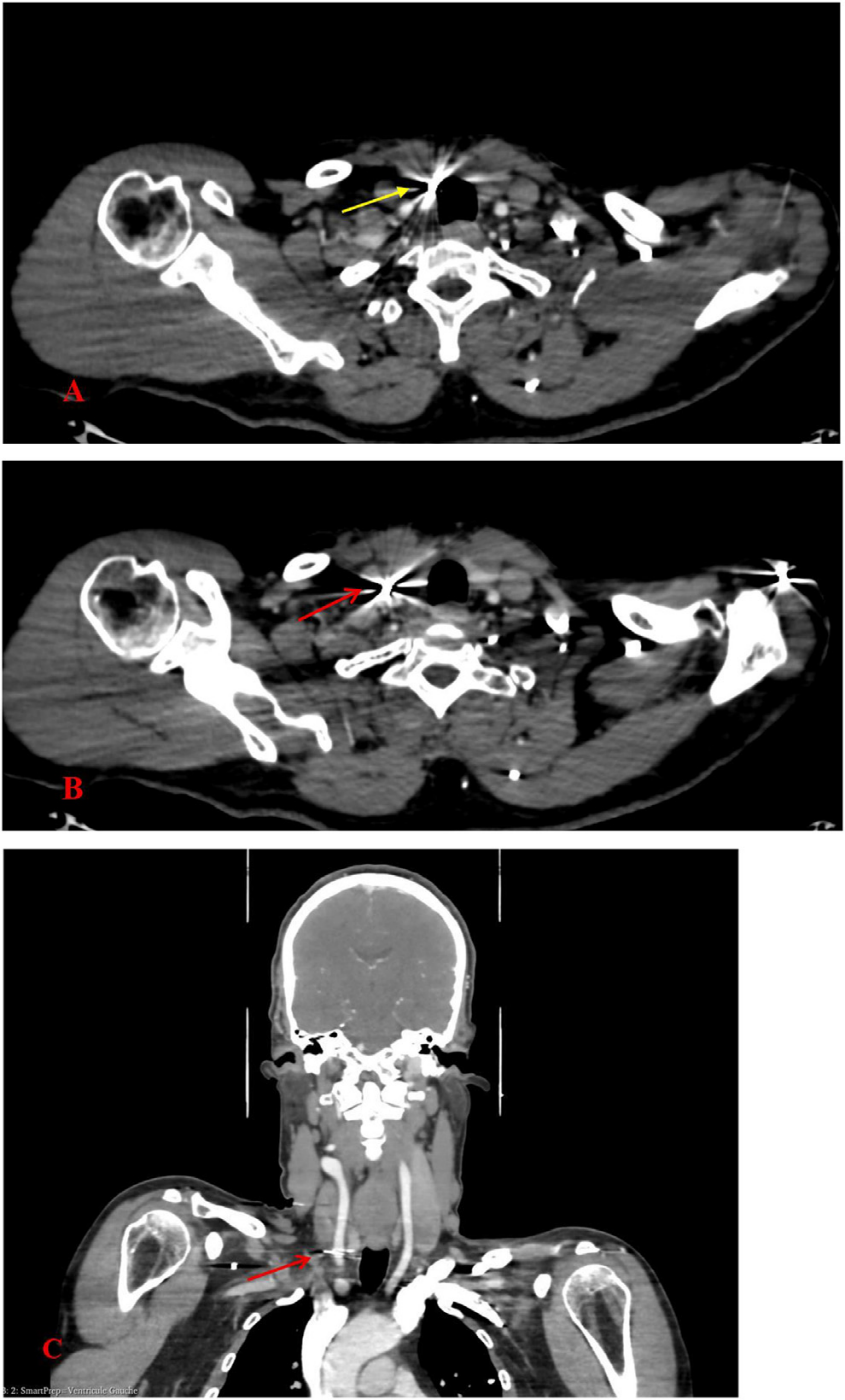
Axial views (A, B) and coronal view (C) showing the stable position of metallic pellets located in the pre‑tracheal region (yellow arrow) and adjacent to the carotid sheath (red arrow), without migration or associated vascular compromise.

Flexible laryngoscopy on day 7 revealed intact tracheal mucosa despite the presence of a pellet adjacent to the wall. A water‑soluble contrast esophagogram showed no esophageal perforation. Serial CTA at 6 hours, day 2, and day 7 demonstrated stable findings (Fig. 6).

The patient was discharged on day 10. At 1‑month follow‑up, clinical examination and control CTA were unremarkable, with complete healing and a favorable outcome. CTA follow‑up was scheduled at 6 and 12 months.

## Discussion

Penetrating ballistic neck injury remains a challenging clinical scenario due to the density of vital structures within a confined anatomical space and the potential for rapid deterioration. Management strategies have evolved from mandatory surgical exploration to a more selective, imaging‑driven approach, in which computed tomography angiography (CTA) plays a key role in diagnosis, risk stratification, and follow‑up. This evolution has been accompanied by a shift from an anatomically zone‑based paradigm to a “no‑zone” approach [2,3]. The present case illustrates how careful integration of clinical assessment and CTA findings can guide safe, non‑operative management in the absence of hard signs of vascular injury.

### Cervical anatomy and zonal classification

The cervical zones were first described by Monson et al. at Cook County Hospital in 1969 [4], and slightly redefined in 1979. This classification, based on the accessibility of vascular and other vital structures (Table 1), historically guided the choice of surgical approach and the degree of urgency.

**Table 1:** Zonal classification of the neck with corresponding major structures.

Zone	Anatomical boundaries	Major structures
I	From the sternal notch to the cricoid cartilage	Origin of the common carotid arteries (CCA), subclavian arteries, brachiocephalic trunk, trachea, esophagus, lung apex
II	Form the cricoid cartilage to the angle of the mandible	Carotid bifurcation, internal jugular vein, vertebral artery, trachea, esophagus
III	Form the angle of the mandible to the base of the skull	Distal segments of the carotid and vertebral arteries, cranial nerves, pharynx

### Incidence and mechanisms

Penetrating ballistic carotid artery injuries are relatively uncommon, representing approximately 3%-11 % of penetrating neck trauma cases. The CCA and internal carotid artery (ICA) are the most frequently involved segments. Ballistic wounds are the leading mechanism, followed by stab wounds and other penetrating injuries. These lesions are associated with significant morbidity and mortality, particularly when accompanied by neurological deficits or massive hemorrhage [5].

### Clinical presentation

• Hard signs: shock, pulsatile bleeding or expanding hematoma, audible bruit or palpable thrill, airway compromise, wound bubbling, subcutaneous emphysema, stridor, difficulty or pain when swallowing secretions, neurological deficits [3].
• Soft signs: odynophagia, dysphagia, minor hemoptysis, nonexpanding hematoma [6].
• Major complication: stroke, which may occur immediately or in a delayed fashion.
• Asymptomatic presentation: possible, as in our patient.

### Imaging

In the absence of hemodynamic instability or active hemorrhage, current literature recommends rapid CTA to define the extent of injury and guide management [7].

CTA demonstrates a specificity and negative predictive value of 97%-100% for penetrating cervical vascular injuries [2,6]. Direct CTA signs of arterial injury include wall irregularity, stenosis, flow interruption, contrast extravasation, pseudoaneurysm, and perivascular hematoma [8].

In this case, CTA provided a comprehensive vascular assessment: it confirmed vessel wall integrity despite projectile contact with the common carotid artery, excluded surgical indications such as active bleeding, pseudoaneurysm, or flow‑limiting stenosis, and served as the baseline for subsequent follow‑up imaging. Nevertheless, interpretation was limited by beam‑hardening artifacts from metallic pellets. These can be mitigated by multiplanar reconstructions, maximum intensity projections, adjustment of window/level settings, and metal artifact reduction algorithms when available. Beyond artifact management, CTA protocols must also account for technical pitfalls such as bolus timing, venous contamination, and motion. Modern recommendations emphasize the use of ≥64‑slice multidetector scanners, thin‑slice reconstructions, and advanced post‑processing (MIP, volume rendering, curved planar reformations) to optimize vascular assessment. CTA also allows detection of more subtle lesions such as vasospasm or intimal flaps, which may otherwise be overlooked. Awareness of these pitfalls and optimization strategies, as highlighted by Kani et al. [9], is essential to avoid misinterpretation and ensure accurate risk stratification in ballistic trauma.

### Surgical vs conservative management

Conservative management of penetrating neck wounds was common in the early 20th century. In 1956, Fogelman and Stewart, based on civilian experience, reported lower mortality with mandatory surgical exploration compared to observation alone [10].

Over time, observational studies suggested that patients without clear clinical signs of vascular or visceral injury could be managed selectively and conservatively, provided close monitoring for up to 48 hours [11,12]. The presence of an associated thoracic injury is not, in itself, an indication for cervical exploration [13].

Historically, penetrating neck injuries were managed according to a rigid zone‑based classification (zones I–III), which often dictated surgical exploration based solely on wound location. Modern practice has shifted toward a more flexible “no‑zone” strategy, in which multidetector CTA plays a central role in diagnosis, risk stratification, and treatment planning [14].

The only randomized trial, conducted by Golueke et al., compared surgical exploration with a conservative approach based on clinical examination and imaging in 160 patients, finding no significant difference in hospital stay, morbidity, or mortality [15]. Initial evaluation should prioritize hemodynamic stabilization according to Advanced Trauma Life Support (ATLS) protocols, with damage control surgery reserved for unstable patients [16].

The latest European Society for Vascular Surgery (ESVS) guidelines endorse conservative management in selected cases, recommending clinical surveillance combined with single antiplatelet therapy for minor (ESVS Grade 1) carotid artery wall injuries from penetrating trauma [17]. This is supported by recent observational data:

▪ In a retrospective cohort of 161 patients, Teixeira et al. demonstrated that a selective surgical approach avoided 59% of unnecessary neck explorations without increasing mortality or morbidity, and reduced mean hospital stay (2 days vs 6 days for mandatory exploration) [18].
▪ Similarly, Zaidi et al., in a prospective series of 46 patients, reported no deaths, fewer complications, and shorter hospitalization with carefully selected conservative management compared to immediate surgical exploration [19].

### Application to the present case

In our patient, the absence of hemodynamic instability, the lack of hard signs, and CTA findings showing pellets confined to the carotid sheath without evidence of intimal injury or hemodynamic compromise supported a conservative approach. Management consisted of close hemodynamic and neurological monitoring, antiplatelet therapy, and scheduled CTA at defined intervals.

CTA proved decisive throughout the patient’s course:

• Diagnosis: confirmed vessel wall integrity despite projectile contact.
• Risk stratification: excluded surgical indications such as active bleeding or flow‑limiting stenosis.
• Follow‑up: documented stability over time, allowing safe discharge.

This case demonstrates that meticulous clinical assessment, combined with high‑quality CTA, can safely guide selective non‑operative management in penetrating ballistic neck trauma without hard signs of vascular injury. When applied to carefully selected patients within a structured multidisciplinary surveillance framework, this approach minimizes unnecessary surgical exploration while maintaining safety. CTA, as an integral component of modern management algorithms, provides rapid, high‑resolution vascular evaluation that supports accurate risk assessment, informed treatment planning, and timely detection of complications. In our patient, adherence to these principles preserved vascular integrity, avoided surgical morbidity, and led to an excellent outcome.

## Conclusion

In hemodynamically stable patients with penetrating ballistic neck injury and minor CCA involvement, selective non‑operative management supported by rigorous CTA‑ follow‑up can be a safe and effective alternative to surgery. CTA serves not only as the diagnostic gold standard but also as a strategic tool for ongoing risk assessment and timely intervention should complications arise.

## Learning points

• CTA is pivotal in the initial assessment, triage, and follow‑up of penetrating neck injuries.
• Conservative management is feasible in carefully selected patients without hard signs of vascular injury.
• Multidisciplinary decision‑making and structured surveillance protocols are essential for patient safety.
• Delayed vascular imaging is necessary to detect late complications such as pseudoaneurysm or thrombosis.

## Patient consent

The authors certify that they have obtained all appropriate patient consent forms. In the form, the patient has given consent for their images and other clinical information to be reported in the journal. The patient understands that names and initials will not be published, and due efforts will be made to conceal their identity, but that anonymity cannot be guaranteed.
